# Foreign Correspondence

**Published:** 1883-06

**Authors:** 


### Foreign Correspondence.

London, May 8th, 1883.

Dear Journal—I am writing this letter in the British Museum Library, and this reminds me of a bit of practical advice for any of your readers who are contemplating a trip to London: It is to bring few or no books with them; either here or at the college libraries, any book wanted for reference can be readily obtained. Heavy baggage is a source of continual vexation and expense to the traveler in Europe.

This grand library deserves a word of mention, for to the mere sight-seer it is one of the sights of the world, and to the scholar it is eminently attractive. Indeed, I pity the man who can stand under this great dome unimpressed. He is surrounded with the records of all that mankind has done, thought, gained, or been, and as it were with the souls of all the wise, great, good, and glorious among the sons of men who have gone before him; as Milton says, "Books are not absolutely dead things, but do contain a progeny of life in them, to be as active as that soul whose progeny they are;" and as we look upon the living men in earnest study of the wisdom here stored up, we are reminded how much the authors of to-day are indebted to the thoughts and work of men whose books live after them. The reading room is circular, surmounted by an immense dome, larger than that of St. Paul's at Rome. The great circle of wall is entirely filled up with books as high as the spring of the dome. Light gilded galleries run around the interior, providing easy access to the books. The light, which is abundant, comes entirely from windows in the dome.

The building contains three miles lineal, of book cases, eight feet high. Assuming them all to be spaced for the average octavo book size, the entire ranges form twenty-five miles of shelves.

Gold and light colors have been employed in the decoration of the interior of the dome and walls, and the effect is exceedingly bright and pleasing; all glare is avoided by admitting the light through opaque glass; at night light is supplied by the electric lamp. The floor is covered with a compound called, here, "kamptulicon," which serves so well in deadening the sound of foot-falls that, although about 300 people are usually present, there is no annoyance from noise.

The catalogue fills two concentric circles of low shelves around a raised enclosure in the centre of the reading room which is occupied by the superintendent and his assistants.

Taking one's seat at the convenient tables which fill the great circle of floor, one can quickly obtain any books desired from the immense collection of two million volumes which the library contains.

Perhaps from its proximity to the museum, Americans who take up their residence in London for a few months seem to gravitate towards Russell square; certain it is that from the numerous boarding houses which occupy the streets leading off that pretty plat of green, daily emerge a colony of Americans in quest of knowledge, mammon or pleasure, as the case may be. So far, we have all escaped arrest if not suspicion of being "Dynamite Fiends," who might mysteriously blow all London to flinders. The "Doctor," who now languishes in a "British Bastile," did not take up his residence in our neighborhood, and does not seem to have interested himself in medical affairs.

The popular alarm has now considerably abated, but after the explosion at the government buildings the wildest notions prevailed as to the destruction which the agents of the "Dynamite Party" might accomplish, and the crazy utterances of the cowardly reprobates who, under the safe protection of the American Eagle made their wild threats, received an amusing attention.

Russell square is also an advantageous location for medical men in that many of the great hospitals are within easy distance. The London Hospital, however, the largest and in many ways the most worthy of frequent visits, is in White chapel, four miles away. The distance, however, should not deter the medical visitor to London from an attendance at its wards.

Every other afternoon Hutchinson, whose reputation here as a surgeon and teacher is unsurpassed, makes his rounds, and I may venture to say that in no English speaking country will so large a number of cases be seen as in his service, which extends usually from 2 to 5.30 P. M.

Andrew Clark, Hughlings Jackson and Sutton, are among the physicians, and as specialists, there are good men in each department. There are over 800 beds in the hospital, and they are all free.

The annual expense of maintenance is $250,000, and this great sum is met almost wholly by subscriptions, there being no endowment as in many of the others. St. Thomas,' Guy's, St. Bartholomew's, own much real estate and other property, which yields so large an income that they are practically independent.

But I promised in my last to say something of the "special" hospitals, some of which are indeed worthy of notice. There is hardly a department of medicine unrepresented by its special hospital.

There are fifteen for diseases of women and lying-in; five each for diseases of the nervous system, chest and throat, eye and ear; two for cancer; seven for diseases of children; one for diseases of the rectum; one for diseases of urinary organs, and four each for opthalmic and orthopædic cases. Some of these hospitals are very large, and the specialist, in any department, will find in London abundance of cases.

For the study of diseases of the chest, one need not ask for anything better than the opportunities to be had at the hospital for consumption, Brompton. This consists of two splendid buildings with a pretty garden attached, and contains 347 beds, which are always full.

Dr. Symes Thompson is the senior physician, and of high reputation here, though I think unknown in America. I can not say that one can learn much as to treatment, for notwithstanding the immense opportunities which such an institution presents for deep and sustained study of a malady which has so far defied our skill, we find here the same old routine of treatment, followed by the same success.

It may perhaps be thought that any advantages to be gained by the superior knowledge of the disease in such a hospital is more than counterbalanced by the evil incurred in bringing together in the same wards a number of persons suffering with phthisis, but the admirable ventilation and construction of the building must reduce that danger to a minimum.

Each ward is provided with a fireplace and windows on three sides, but independently of these 4,000 cubic feet of air to each patient is hourly introduced, a uniform temperature being obtained by causing the air to pass over coils of pipe containing hot water. The vitiated air is drawn off through extracting flues, built in the wall, which lead into large air ducts beneath the roof, terminating in four towers heated by steam coils forming the exhausting chamber. This plan of ventilation gives entire satisfaction, and has been adopted after a long trial of other systems.

You will not wonder at my surprise at learning that during the last year 1,044 gallons of cod liver oil had been prescribed and dispensed, and in addition 825 gallons of various alcoholic stimulants. It was a relief to find, however, that this had not all been swallowed by the poor in-patients, the out-patients, of which there are nearly 8,000 annually, taking their quota.

Every afternoon from 12.30 to 4 o'clock, out-patients are seen; there is always a crowd of them; and every variety of lung and heart disease comes under one's stethoscope.

When one knows the miserable condition in which the poor of London live, the vitiated atmosphere, over-crowded and ill-constructed dwellings and work shops, located in narrow lanes far from the parks and other breathing places, wonder at the prevalence of phthisis and other diseases, ceases.

The mortality from phthisis is fearful enough, the registrar general's last report showing that of the 60,000 deaths occurring in the metropolis, about 7,500, or one-eighth of the total mortality at all ages, and a little less than one-fifth of the mortality of adults, arises from this disease.

St. Mark's Hospital, for fistula and other diseases of the rectum, is, I am told, the only hospital in the world devoted solely to the treatment of this class of diseases. Its wards are commodious and always well filled with patients, and a great number of out-patients are also treated. There are three excellent surgeons connected with the hospital. Of these Allingham is one. His name is so well known through the immense circulation which his book on diseases of the rectum has attained, that he always attracts a large number of medical men to witness his operations.

In his work, after a review of the various methods of operation for internal hemorrhoids, he strongly recommends operating by the ligature, which he says is the "safest, easiest and best." I was surprised, therefore, to see the ligature discarded and the clamp used in every case.

In explanation he said that it was only recently that he had given the preference to the clamp, or rather, *crusher*, which he now employs. When the last edition of his book appeared, he had used it less than fifty times, and on that experience did not feel justified in unqualifiedly commending it. He had now employed it in over 300 cases without a death or even a single complication.

In using it, the patient being fully anæsthetized, he always completely dilates the sphincter muscles. The hemorrhoids are then taken up, one by one, drawn down and separated from its connection with the muscular and submucous tissues upon which it rests, with a pair of strong scissors.

The cut is made where the skin meets the mucous membrane, and is carried up the bowel and parallel to it, so that the pile is left connected by an isthmus of vessels and mucous membrane only. The crusher is then applied, screwed up tightly, and allowed to remain one minute. He never has hemorrhage. The after-treatment is as described in his book.

The one hospital devoted to the treatment of genito-urinary diseases is called St. Peter's Stone Hospital. This institution has lately taken possession of a new and fine building, and seems to be well supported. Notwithstanding its name, operations for stone are not very frequent; when they do occur lithotrity is employed, by preference in all suitable cases. To those interested in the study of the treatment of strictures, this hospital is attractive, as a greater number of cases can be seen here than elsewhere in London. Coulson and Bruce Clark are the chief surgeons. In all cases of stricture, simple dilatation is always first tried; the flexible French bougie with tapering and bulbous ends is always employed. Next to this in favor comes continuous dilatation, followed, if necessary, by internal urethrotomy.

A modification of Maisoneure's urethrotome is employed, but my prejudice against that instrument is rather increased than diminished, by observing the results obtained by its use, even in such experienced hands. With Sir Henry Thompson's and other modifications of Civiale's instrument, the surgeon can control and regulate its powers according to the necessities of the case, and thus ensure a complete division of the stricture. As Sir Henry Thompson says: "If an incision of a stricture is required at all, it is essential that the whole of the obstructing fibres be divided. The cases of relapse are those in which the incision failed to cut the most elastic and yielding fibres, which at no distant time show their presence and reproduce contraction."

Divulson I have not seen performed here at all, Thompson limits its application to stricture within the bulb. At this hospital one sees also an immense number of cases of syphilis, gonorrhœa, etc., but there is nothing noticeable in the treatment, the ordinary routine being followed.

To those interested in dermatology, London provides admirable opportunities.

In addition to the four "Skin Hospitals," every general hospital has its special department for diseases of the skin, and one may, if so disposed, spend every afternoon and two entire mornings oł each week in seeing such cases, and observing the treatment employed by men of great experience. Of the teachers here, I am disposed to rank Liveing first, though Crocker, Sangster, Warren Tay, S. MacKenzie, Fox and Morris, are all good. MacKenzie, who is at the London Hospital, always has the largest number of cases, about two hundred, usually; but this is a disadvantage, because to get through in any reasonable time he is compelled to "rush" his patients, and there is little time for observation of any case which interests one.

It is his custom, in cases of alopecia areata, to paint on cantharidal collodion, full strength, to every bald spot. It does not cause as much pain as one would expect, rarely blistering very severely, but I cannot learn that he has any better results than those who employ milder stimulation. Psoriasis is very common here, coming next in point of frequency to eczema. Of the vegetable parasites, the trichophyton is most abundant. Tinea Tonsurans is surprisingly common, one of the results, as it is said, of the operation of the new law making the attendance of children at school compulsory. Pityriasis Versicolor and Favus are but seldom seen. Pediculi and even acari would seem to find an almost undisturbed home on the persons of a certain class of English people; at least they endure their presence till in a frightful condition before applying for even free medical advice.

At the last meeting of the London Dermatological Society, Mr. Hutchinson presented a case of Rhinoscleroma which attracted much attention, as the disease had not been hitherto seen in England; Hebra and Kaposi report, in all, thirty-five cases. It has, I think, never been seen in America. It is a new growth of the skin and mucous membrane of the nose and contiguous parts, characterized by the formation of circumscribed nodules, papules and tubercles, painful upon pressure, slowly enlarging and becoming, finally, as dense as ivory. The disease, in this case, began two years ago as a papule upon the septum of the nose without inflammatory symptoms. It now involves the whole of the nose and upper lip and gum, and corresponds in every respect to the description given by Kaposi, both in its progress and present appearance. Unfortunately it is a disease in which treatment has been found of little avail. Excision by the knife and destruction by caustics have been only of temporary benefit, as the growth is soon reproduced.

Dr. S. MacKenzie presented a case for diagnosis, a woman whose face was covered with innumerable minute papilliform excrescences. It was generally recognized as a case of verruca plana.

Dr. Sangster showed a case of tinea unguim, the microscope clearly showing the presence of the trycophyton in the scrapings from the nail. Dr. Fox brought forward a patient whose nails were similarly affected.

Dr. Crocker presented a case of diffuse generalized Telangiectasis. In happy contrast, Dr. Fox showed a case of capillary nævi, thelesions appearing over a considerable portion of chest and, limbs. A case of lupus, in which the diagnosis was not easy, and several other more common cases, were also presented.

The meetings of the medical societies here are usually well attended. The discussions are not as well sustained as one would expect from the vast opportunities for observation and study which the medical men of London have. At some of the societies the members usually appear in full evening dress, and at all of them coffee, tea and other refreshments are served. During the winter months, several interesting courses of lectures have been delivered by eminent men before the Royal College of Physicians. To one accustomed to republican simplicity it is odd enough to see the formalities which attend the delivery of these lectures. At the appointed hour a procession of the "fellows" of the society make their appearance in the amphitheatre, preceded by a stalwart servant in livery bearing aloft an immense brass mace surmounted by a crown. The lecturer comes in at the end of the procession, wearing a bright red gown. When all have taken their places, the mace is laid upon a table in front of the lecturer, who then proceeds with his discourse. I have had the pleasure of hearing several of these lectures, but as they have been published in the *Lancet* and other weeklies, I need not further refer to them; indeed, I feel that I already ought to apologize for the length of this letter. A. R. D.

